# Molecular characterization and sensitivity to demethylation inhibitor fungicides of *Aspergillus fumigatus* from orange-based compost

**DOI:** 10.1371/journal.pone.0200569

**Published:** 2018-07-12

**Authors:** Massimo Pugliese, Slavica Matić, Sanila Prethi, Ulrich Gisi, Maria Lodovica Gullino

**Affiliations:** 1 AGROINNOVA–Centre of Competence for the Innovation in the Agro-Environmental Sector, Turin University, Largo P. Braccini 2, Grugliasco, Turin, Italy; 2 Agricultural, Forestry and Food Sciences Department (DISAFA), Turin University, Largo P. Braccini 2, Grugliasco, Turin, Italy; 3 Alexander Technological Institute of Thessaloniki, Sindos, Thessaloniki, Greece; Leibniz-Institut fur Naturstoff-Forschung und Infektionsbiologie eV Hans-Knoll-Institut, GERMANY

## Abstract

*Aspergillus fumigatus*, the causal agent of human aspergilloses, is known to be non-pathogenic in plants. It is present as saprophyte in different types of organic matter and develops rapidly during the high-temperature phase of the composting process. Aspergilloses are treated with demethylation inhibitor (DMI) fungicides and resistant isolates have been recently reported. The present study aims to estimate the abundance, genetic diversity and DMI sensitivity of *A*. *fumigatus* during the composting process of orange fruits. Composting of orange fruits resulted in a 100-fold increase in *A*. *fumigatus* frequency already after 1 week, demonstrating that the degradation of orange fruits favoured the growth of *A*. *fumigatus* in compost. Most of *A*. *fumigatus* isolates belonged to mating type 2, including those initially isolated from the orange peel, whereas mating type 1 evolved towards the end of the composting process. None of the *A*. *fumigatus* isolates expressed simultaneously both mating types. The 52 investigated isolates exhibited moderate SSR polymorphisms by formation of one major (47 isolates) and one minor cluster (5 isolates). The latter included mating type 1 isolates from the last sampling and the DMI-resistant reference strains. Only few isolates showed *cyp51A* polymorphisms but were sensitive to DMIs as all the other isolates. None of the *A*. *fumigatus* isolates owned any of the mutations associated with DMI resistance. This study documents a high reproduction rate of *A*. *fumigatus* during the composting process of orange fruits, requesting specific safety precautions in compost handling. Furthermore, azole residue concentrations in orange-based compost were not sufficient to select *A*. *fumigatus* resistant genotypes.

## Introduction

The production of compost recently gained much importance in the EU as a waste management process. Composting reduces the volume of waste going to landfills, and the related CH_4_ and CO_2_ emission due to organic material degradation [1]. Composting process allows to upcycle a variety of organic materials and biowastes originating from different sources. The process begins with a thermophilic phase, when temperature reaches 55–65° C, followed by a maturing and mesophilic stage when the temperature declines and the material stabilizes. Reaching a temperature of 55° C for at least 3 days is an important step to guarantee sanitization and microbial safety of compost products [2]. It is specifically recommended by compost quality regulations applied in several EU countries.

The opportunistic fungal pathogen *Aspergillus fumigatus* (Fresen., syn. *Neosartorya fumigata*, O'Gorman, Fuller and Dyer), causal agent of the “aspergilloses” in immunocompromised human hosts, is known to be present in different types of compost [3–6]. It can survive successfully under a wide range of environmental conditions, particularly due to its capacity to resist high temperatures (thermo-tolerant).Consequently, it is prevalent during the high-temperature phase of the composting process and it can be present in compost at concentrations of up to 10^6^−10^7^ cfu/g dw [5,7,8]. The spores are released to the air during composting activities such as heat turning, reaching concentrations of 10^4^−10^7^/m^3^ air [9,10]. Although it can be present in relatively high concentrations, the current hygienic EU regulations for compost do not require an estimation of its presence but rather seek for the absence or limited presence of other human pathogens such as *Salmonella* spp. and *Escherichia coli* [1].

Demethylation inhibitor fungicides (DMIs, chemically named also as ‘azoles’) are the principal antifungal compounds used in the treatment (prophylactic and curative) of human and animal diseases caused by *A*. *fumigatus* [11]. DMIs inhibit the enzyme lanosterol 14-*α* demethylase (encoded by *cyp51* gene, A and B paralogues), involved in ergosterol biosynthetic pathway of fungi [12,13]. Chemically related molecules have also been used for fruit pre- and post-harvest treatments and application of field crops in agriculture for more than 30 years [14,15]. Resistance to medical triazoles (e. g. itraconazole) is well-known since the 1990s and associated with several mutations in the *cyp51A* gene of *A*. *fumigatus* occurring in patients having been treated with these antifungal compounds [16]. Recently, triazole resistant strains have been isolated also from patients not previously treated with triazoles, suggesting that they have picked up air spores originating from any kind of DMI treated material in agriculture and general environment [3,17]. Since compost is one of the most important environmental sources for *A*. *fumigatus* [14], DMI resistant strains might have their origin from this substrate contributing to human health risks [3]. Recently, many azole resistant strains of *A*. *fumigatus* containing a new resistance mutation with clinical relevance were found in compost produced from conventional flower bulbs leftovers [6]. However, it is still not clear whether these mutants evolved during the composting process (e.g. by DMI residues) or were selected by earlier DMI treatment of plant material which was then used to produce the compost pile. Indeed, we previously investigated the presence of *A*. *fumigatus* isolates in commercial composts produced from different wastes, with different composting methods in different locations (Italy, Spain, Hungary, The Netherlands, Germany and United Kingdom): *A*. *fumigatus* was present in all composts but not a single isolate shown mutations for DMI resistance [4,5].

The aim of this study was to estimate the presence and abundance of *A*. *fumigatus* during the composting process of orange fruits, commonly treated with post-harvest fungicides like imazalil (a DMI fungicide), and to evaluate whether this biowaste may be considered as a “hot spot” for DMI resistance evolution. Furthermore, the genetic diversity of isolates, their sensitivity to DMIs and the molecular characterization of the coding part of the *cyp51A* gene were assessed, in order to identify possible mutations occurring during the composting process. Our hypothesis was that the composting process of orange fruits would allow *A*. *fumigatus* to multiply and reproduce, but that the low azole concentrations present in the orange peel would not select for azole-resistant *A*. *fumigatus* genotypes.

## Materials and methods

### Collection of oranges samples, identification and quantification of *A*. *fumigatus* isolates

Oranges samples (around 300 orange fruits) have been collected in Ivrea among leftovers from the local carnival. Oranges were specifically produced for the carnival in the area of Corigliano Calabro (Southern Italy, GPS coordinates: 39°40'21.7"N 16°25'42.0"E), and not used for food purposes. The oranges were routinely treated by imazalil in order to prevent the development of post-harvest fungal diseases, with a maximum residue level <5 mg per kg of orange fruits according to EU regulation (http://ec.europa.eu/food/plant/pesticides/eu-pesticides-database/public/?event=homepage&language=EN). Twenty percent of the oranges have been examined for the presence of *A*. *fumigatus*, the rest was delivered to an industrial composting plant located in Piedmont. Isolation and identification of *A*. *fumigatus* was carried out following the protocol by Franceschini *et al*. [4]. One g of three subsamples (orange peel) was added to 9 ml Ringer solution and shaken for 1 hour at 100 rpm. One hundred μl of suspension and serial dilutions of it were plated on 20 ml of potato dextrose agar (PDA, Merck®, Darmstadt, Germany) containing streptomycin (50 mg/l, Applichem, Darmstadt, Germany), in triplicates. Plates were incubated at 42° C for 3–5 days.

Typical powdery green-grey colonies of two isolates (AR4.9 and AR6.1) from two orange fruits were purified, and identified by macro and micro-morphological characteristics. The plates were incubated at 50° C with the aim to distinguish *A*. *fumigatus* from other *Aspergillus* species. Colonies capable to grow at 50° C were counted on all plates, multiplied by the dilution factor and converted to one gram of dry weight (dw) of the orange peel. Monoconidial cultures obtained from each isolate were stored in 30% glycerol solution at -80° C.

### Preparation of orange-based compost

The oranges were composted using a bench-scale system consisting of 3.5-L PVC plastic units incubated at 50° C for 1 month starting from March 6^th^ 2017. To do so, four kg of oranges were crushed and added to each unit embedded in a plastic bag to avoid external contaminations. The composting process started with a first phase of active degradation lasting nearly 7 weeks, followed by another 4 weeks of maturation phase at a pH > 7. Compost samples were collected during 10 sampling dates (86 days in total), at a 7–14 days interval, and the composting material was well mixed every time (Table 1).

**Table 1:** List of *A*. *fumigatus* isolates selected for sensitivity assays with accession numbers and mating type.

Code	Sampling date	Accession no. (ITS)	Accession no. (*cyp51A*)	Mating type	
				1	2
AR4.9	28 February 2017		MH026061		+
AR6.1			MH026062		+
502.1_13.3	13 March 2017	MG970365	MH026063		+
502.2_13.3		MG970366			+
502.3_13.3		MG970367			+
502.4_13.3		MG970368			+
502.5_13.3		MG970369			+
502.1_20.3	20 March 2017	MG970370			+
502.2_20.3		MG970371			+
502.3_20.3		MG970372			+
502.4_20.3		MG970373			+
502.5_20.3		MG970374	MH026064		+
502.1_27.3	27 March 2017	MG970375			+
502.2_27.3		MG970376			+
502.3_27.3		MG970377			+
502.4_27.3		MG970378			+
502.5_27.3		MG970379	MH026065		+
502.1_3.4	3 April 2017	MG970380			+
502.2_3.4		MG970381			+
502.3_3.4		MG970382	MH026066		+
502.4_3.4		MG970383			+
502.5_3.4		MG970384			+
502.1_10.4	10 April 2017	MG970385	MH026067		+
502.2_10.4		MG970386			+
502.3_10.4		MG970387			+
502.4_10.4		MG970388			+
502.5_10.4		MG970389			+
502.1_18.4	18 April 2017	MG970390	MH026068		+
502.2_18.4		MG970391			+
502.3_18.4		MG970392			+
502.4_18.4		MG970393			+
502.5_18.4		MG970394			+
502.1_24.4	24 April 2017	MG970395			+
502.2_24.4		MG970396	MH026069	+	
502.3_24.4		MG970397	MH026070		+
502.4_24.4		MG970398	MH026071		+
502.5_24.4		MG970399			+
502.1_2.5	2 May 2017	MG970400	MH026072		+
502.2_2.5		MG970401			+
502.3_2.5		MG970402			+
502.4_2.5		MG970403			+
502.5_2.5		MG970404			+
502.1_8.5	8 May 2017	MG970405	MH026073		+
502.2_8.5		MG970406			+
502.3_8.5		MG970407			+
502.4_8.5		MG970408			+
502.5_8.5		MG970409			+
502.1_24.5	24 May 2017	MG970410		+	
502.2_24.5		MG970411	MH026074	+	
502.3_24.5		MG970412	MH026075	+	
502.4_24.5		MG970413	MH026076	+	
502.5_24.5		MG970414		+	

### Identification and quantification of *A*. *fumigatus* isolates

Fifty *A*. *fumigatus* isolates (5 isolates per each sampling date) were collected during the composting process and identified as described above (Table 1). For isolation of *A*. *fumigatus* from compost, we used a selection medium without azoles in order to pick up isolates with reduced sensitivity to DMIs as well as fully sensitive isolates for further *in vitro* sensitivity assays.

Fifty isolates from compost together with the two isolates from intact orange fruits were used subsequently for molecular identification using internal transcribed spacer (ITS) region, microsatellite analysis, sensitivity assays to DMIs, and molecular characterization of the *cyp51A* gene. Fungal DNA was extracted by the EZNA® Fungal DNA extraction kit (Omega Bio-Tek, Darmstadt, Germany) following the manufacturer’s instructions. The amplification of the ITS region was performed as described by White *et al*. [18], and the sequences of the ITS amplicons were used for molecular identification.

### Mating types identification

Mating type of *A*. *fumigatus* isolates was determined by using multiplex PCR according to Paoletti *et al*. [19]. Multiplex PCR assay included *MAT-1* specific primer (AFM1, 5′-CCTTGACGCGATGGGGTGG- 3′), *MAT-2* specific primer (AFM2, 5′-CGCTCCTCATCAGAACAACTCG-3′), and AFM3 common primer (5′-CGGAAATCTGATGTCGCCACG-3′) [19]. Reaction volumes (25 μl) contained 10 ng DNA, 1× PCR buffer, 1.4 mM MgCl_2_, 0.4 μM primer AFM1, 0.4 μM primer AFM2, 0.8 μM AFM3, 0.2 mM dNTPs, and 1 U Taq DNA polymerase (Qiagen, Hilden, Germany). PCR cycling conditions consisted of 5 min at 95° C of initial denaturation step, followed by 35 cycles of 30 s at 95° C, 30 s at 60° C and 1 min at 72° C, and a final extension step of 5 min at 72° C. Five microliters of each PCR product were electrophoresed on a 1.2% agarose gel pre-stained with RedGel (Biotium, Hayward, CA, USA) and visualized under UV light. The mating type of each isolate was assigned due to the size of the amplicon: 834 bp (MAT-1), and 438 bp (MAT-2).

### Genetic diversity assessment of *A*. *fumigatus* isolates

Six microsatellite regions (STR*Af* 3A, 3B, 3C, and STR*Af* 4A, 4B, 4C) of 52 *A*. *fumigatus* isolates were amplified using the set of primers described by Valk *et al*. [20]. Four reference environmental isolates (provided by B. Fraaije, Rothamsted Research, Harpenden, UK) were also included in the analysis: wild-type isolate (WT), and resistant isolates (TR_34_+L98H from UK, TR_46_+Y121F+T289A from UK and TR_34_+L98H from NL). The PCR mixture (20 μl) contained 1 ng of genomic DNA, 1× PCR buffer, 1 μM of each primer, 0.2 mM dNTPs, 2.0 mM of MgCl_2_, and 1 U Taq DNA polymerase (Qiagen). PCR conditions were as follows: initial denaturation at 95° C for 10 min, followed by 30 cycles of denaturation at 95° C for 30 s, annealing at 60° C for 30 s and extension at 72° C for 1 min, and final extension at 72° C for 10 min. STR*Af* 3B and STR*Af* 4B microsatellite loci were amplified using an annealing temperature of 65° C. Amplified DNA fragments were separated on 3% MetaPhor® agarose gel (Lonza, Rockland, USA), and then analyzed by PyElph 1.4 software [21]. The calculation of genetic distance between isolates was performed by GenAlEx 6.502 software [22,23]. Cluster analyses of polymorphism detected by six SSR markers were performed using the Unweighted Pair Group Method with Arithmetic Averages (UPGMA) of the MEGA 7 software [24].

### *In vitro* sensitivity testing of *A*. *fumigatus* isolates to DMIs

Fifty-two *A*. *fumigatus* isolates were tested for *in vitro* sensitivity to the DMIs by using one post-harvest and veterinary medical DMI (imazalil, syn. enilconazole) and two human medical DMIs (posaconazole and voriconazole). EUCAST protocol [25] was applied with slight modifications (incubation temperature at 37° C, and visual reading of results using turbidity scale) following the FRAC standard methods by *in vitro* assays for plant pathogens on microtiter plates (http://www.frac.info/monitoring-methods). Four environmental isolates were additionally tested as the reference isolates (WT isolate and 3 resistant isolates as indicated above).

Imazalil (PESTANAL® analytical standard, Sigma-Aldrich), voriconazole, and posaconazole (VETRANAL™ analytical standard, Sigma-Aldrich) were tested using 5-fold serial dilutions (from 50 to 0.08 mg/l), in two replicates. RPMI 1640 medium (with L-glutamine, Sigma-Aldrich) containing 2% glucose, 3-(N-morpholino) propanesulfonic acid (MOPS; final concentration of 0.165 mol/l, pH 7.0), and each specific fungicide was loaded (100 μl/well) into flat-bottom Nunc™ 96-well microplate (Thermo Fisher Scientific, Wilmington, USA). One hundred μl of *A*. *fumigatus* spore suspension (2–5 × 10^5^ conidia/ml) was then added to each well. Appropriate controls (without fungicide or without *A*. *fumigatus*) were also included. The microplates were incubated for 48 hours at 37° C. Mycelial growth of all isolates was visually assessed [25] by grading turbidity scale (0–5) with 0 referring to optically clear and 5 indicating no reduction in turbidity compared with that in the control (fungicide-free) well.

The percent growth inhibition (GI) was calculated as: % GI = (Gc–Gf / Gc) x 100, where ‘Gf’ indicates the growth percentage at each fungicide concentration, and ‘Gc’ refers to the growth control. EC_50_ values (or concentrations causing 50% growth inhibition) were calculated using a ‘log/logit dose response’ parameter of the GraphPadPrism® software (version 7.02; La Jolla, CA, USA). The log fungicide concentration vs. normalized response-variable (GI) procedure using a logistic regression was calculated as: Y = Bottom + (Top-Bottom) / {1+10 [(LogEC_50_-X) × HillSlope]}, with Y indicating the response, X referring to the fungicide concentration, Top and Bottom indicating the plateaus in the Y axis units, and Hillslope refering to the steepness of the curve [26]. EC_50_ values were used rather than MIC values due to their very precise calculation of the dose—response relationship (see FRAC guidelines).

The sensitivity distribution or frequency distribution of isolates at certain EC_50_ level was determined for imazalil, posaconazole and voriconazole using the ‘box-and-whiskers’ plot method. This method provides the variability, shape, and characteristic distribution values through the maximum and minimum values (‘whiskers’), the interquartile range (the differences between the various interquartiles), box (50% of population), the median (central line) value, and the average value [27].

### Molecular characterization and sequence analyses of the *cyp51A* gene

The full *cyp51A* gene of 52 *A*. *fumigatus* isolates was amplified according to Snelders *et al*. [28] using the primers P450-A1 (5’-ATGGTGCCGATGCTATGG-3’) and P450-A2 (5’-CTGTCTCACTTGGATGTG-3’). The PCR cycling conditions consisted of an initial denaturation of 5 min at 95° C, followed by 40 cycles at 94° C for 30 s, 58° C for 45 s, and 72° C for 2 min, and a final extension at 72° C for 7 min.

The ITS and *cyp51A* PCR products were sequenced at BMR Genomics (Padua, Italy). The GenBank accession numbers of the ITS and *cyp51A* sequences are reported in Table 1. BLAST searches of obtained sequences were performed against the GenBank databases at NCBI. Multiple sequence alignment was performed by Vector NTI Advance 11 software (InforMax, North Bethesda, Maryland, USA) using the Clustal W algorithm [29]. The *cyp51A* sequence of the WT isolate (accession no. AF338659), sensitive compost isolates [4,5] and resistant clinical and environmental isolates (ITZ.86, 11_0087A, 14, and 98) [28,30–32] were included in the analyses. Phylogenic analyses were carried out using MEGA 7 software, creating the neighbour-joining (NJ) trees with 1000 bootstrap replications.

## Results

### Morphological and molecular identification of *A*. *fumigatus* on orange peel

Out of 60 orange fruits, two *A*. *fumigatus* isolates (AR4.9 and AR6.1) were detected on the peel of two different asymptomatic orange fruits, and identified morphologically (frequency of 3.3% on fruits) corresponding to a concentration of 0.01 to 0.04 × 10^3^ cfu *A*. *fumigatus* per g of dw of orange peel. The two isolates AR4.9 and AR6.1 were confirmed as *A*. *fumigatus* by molecular characterization on the basis of ITS sequencing (GeneBank Accession Nrs. MG976901 and MG976902).

### Identification and quantification of *A*. *fumigatus* isolates in orange-based compost

**Fig 1:**
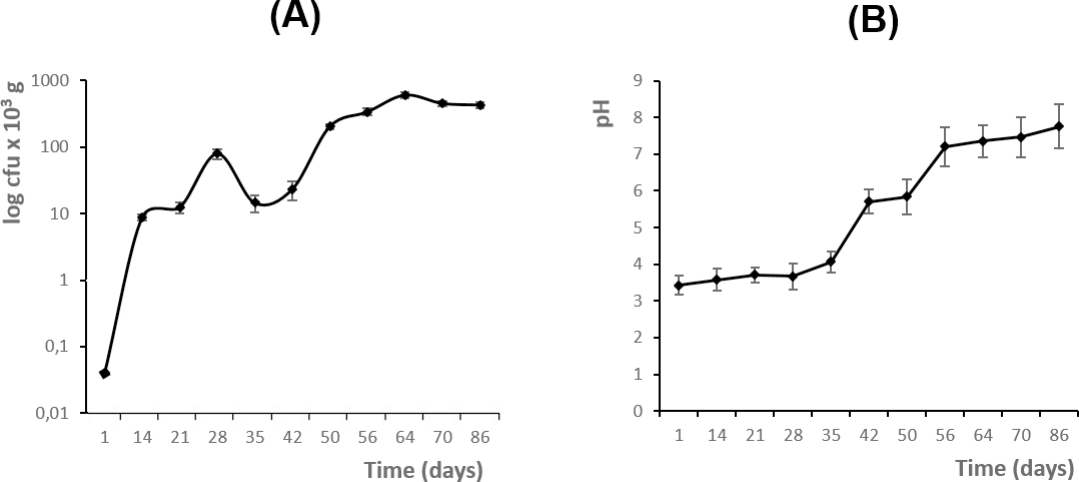
**Abundance of *Aspergillus fumigatus* (A) and measured pH values of orange-based compost (B) during the composting process.** Both analyses were done during 10 sampling periods. Points represent mean values, and error bars show standard deviations.

In total, 50 orange-based compost isolates of *A*. *fumigatus* from different composting phases were collected and identified on the basis of macro and micro morphological observations. During each sampling date, one compost sample was collected and divided into three subsamples. Five randomly selected colonies per each sample (including all three subsamples) were confirmed as *A*. *fumigatus* by ability to grow at 50° C and sequencing ITS amplicons (Table 1). The abundance of isolates was measured and expressed as cfu/g dw of compost (Fig 1A). The values at the beginning of the composting process were at 8.8 × 10^3^ cfu/g, increased more or less steadily and reached highest concentrations on a plateau level at 430.7 to 605.7 × 10^3^ cfu/g at the last three dates of sampling (Fig 1A).

In addition, the pH of compost samples was measured; it was 3.6 at the beginning and increased steadily during the composting process reaching a value of 7.8 at the end (Fig 1B). The fungus was able to grow under both acidic and slightly basic conditions, with the highest abundance at neutral to slightly basic conditions during the second half of composting when the composting process was almost accomplished.

### Mating types identification

Out of 52 *A*. *fumigatus* isolates, six expressed mating type 1 (12%) and 46 mating type 2 (88%) (S1 Fig and Table 1). No isolate showed amplification of both mating-type bands. Interestingly, all five isolates originating from the last (10^th^) sampling were of mating type 1, the other mating type 1 isolate came from the seventh sampling period.

### Genetic diversity of *A*. *fumigatus* isolates

**Fig 2:**
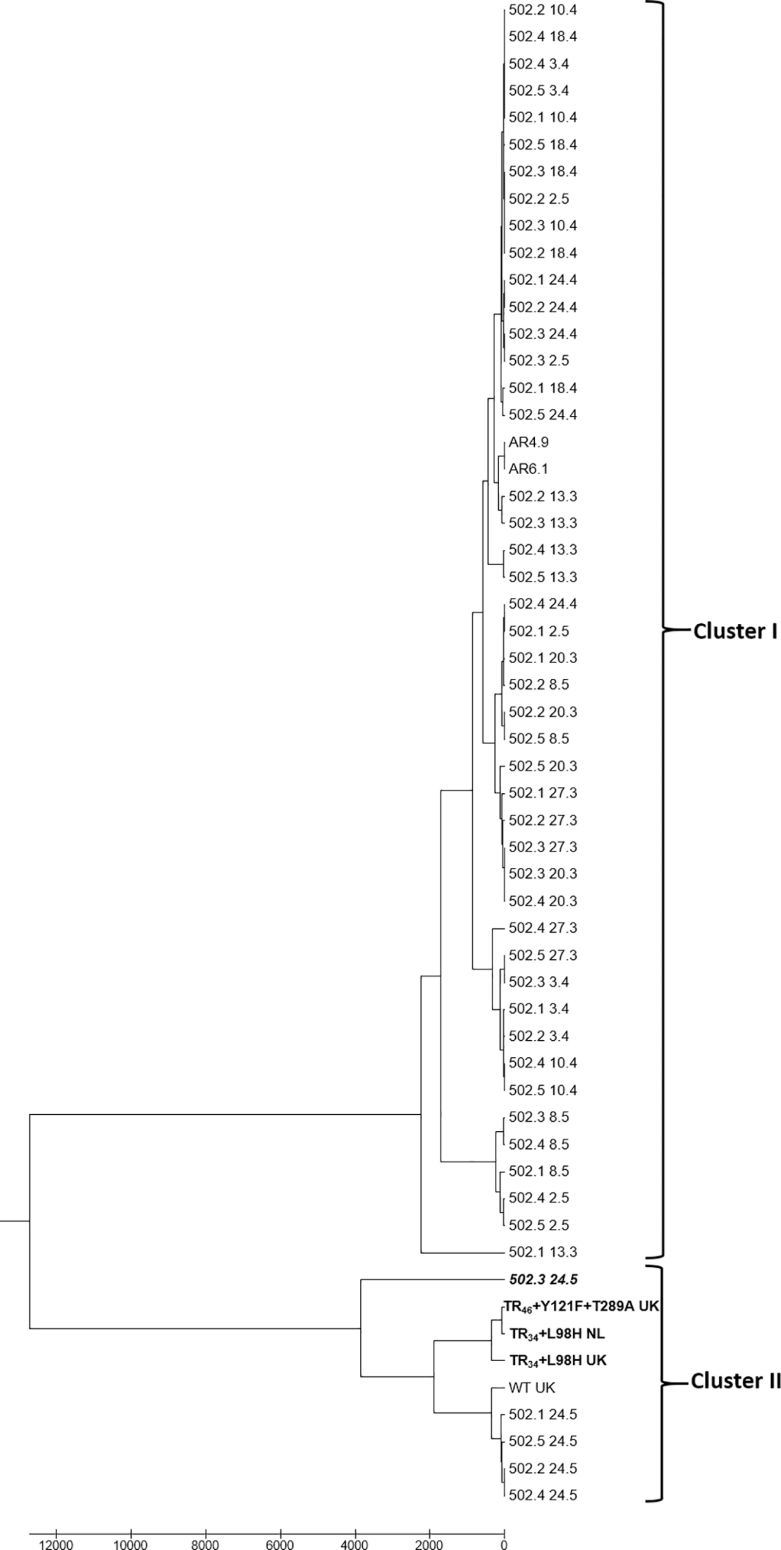
SSR genetic diversity of 52 *A*. *fumigatus* isolates from oranges and orange-based compost. Three reference DMI resistant isolates are indicated in bold, and one compost isolate (502.3_24.5) with reduced sensitivity to voriconazole and posaconazole, in italics. WT, wild-type reference isolate; UK, United Kingdom; NL, The Netherlands.

Based on six SSR markers, the genetic diversity within the population of 50 *A*. *fumigatus* isolates from orange-based compost and 2 isolates from orange fruits was moderate, with two main clusters (Fig 2). The first cluster contained most (47) of the isolates and all isolates that were sensitive to DMIs. The second cluster included 3 reference DMI resistant isolates and the orange-based compost isolate 502.3_24.5 that showed reduced sensitivity to voriconazole and posaconazole (Table 2). Three resistant reference isolates were in a separate sub-cluster, while the orange-based compost isolate 502.3_24.5 was distant from the other isolates. Interestingly, all *A*. *fumigatus* isolates from the last sampling date and the reference wild-type isolate were also included in the second cluster. Moreover, the sub-clusters in both clusters contained mainly isolates from the same or close sampling periods.

**Table 2:** Sensitivity to three DMI fungicides (EC_50_) of *Aspergillus fumigatus* isolates from orange fruits and orange-based compost.

No	Isolate	Voriconazole	Posaconazole	Imazalil	Sample/origin
1	AR4.9	0.11	<0.01	<0.01	Orange/I
2	AR6.1	0.13	<0.01	<0.01	Orange/I
3	502.1_13.3	0.62	<0.01	0.08	Orange compost/I
4	502.2_13.3	0.38	0.07	0.03	Orange compost/I
5	502.3_13.3	0.22	0.48	0.08	Orange compost/I
6	502.4_13.3	0.41	0.04	0.08	Orange compost/I
7	502.5_13.3	0.26	<0.01	0.09	Orange compost/I
8	502.1_20.3	1.21	0.32	0.08	Orange compost/I
9	502.2_20.3	1.59	0.16	0.08	Orange compost/I
10	502.3_20.3	1.37	0.18	<0.01	Orange compost/I
11	502.4_20.3	1.70	0.24	0.09	Orange compost/I
12	502.5_20.3	1.59	0.19	0.03	Orange compost/I
13	502.1_27.3	1.33	0.03	<0.01	Orange compost/I
14	502.2_27.3	1.37	<0.01	<0.01	Orange compost/I
15	502.3_27.3	1.37	0.02	0.08	Orange compost/I
16	502.4_27.3	0.23	0.06	<0.01	Orange compost/I
17	502.5_27.3	1.33	<0.01	0.03	Orange compost/I
18	502.1_3.4	1.50	0.03	0.08	Orange compost/I
19	502.2_3.4	1.33	0.07	0.08	Orange compost/I
20	502.3_3.4	1.32	0.29	0.03	Orange compost/I
21	502.4_3.4	0.71	0.22	<0.01	Orange compost/I
22	502.5_3.4	0.86	0.62	<0.01	Orange compost/I
23	502.1_10.4	0.43	0.11	<0.01	Orange compost/I
24	502.2_10.4	1.54	0.33	0.07	Orange compost/I
25	502.3_10.4	0.14	0.27	0.08	Orange compost/I
26	502.4_10.4	0.43	0.72	0.08	Orange compost/I
27	502.5_10.4	0.41	0.47	0.03	Orange compost/I
28	502.1_18.4	0.13	0.36	0.08	Orange compost/I
29	502.2_18.4	1.36	0.41	0.08	Orange compost/I
30	502.3_18.4	1.20	0.20	0.09	Orange compost/I
31	502.4_18.4	0.63	0.68	0.08	Orange compost/I
32	502.5_18.4	0.57	<0.01	<0.01	Orange compost/I
33	502.1_24.4	0.23	0.43	<0.01	Orange compost/I
34	502.2_24.4	1.59	0.12	0.08	Orange compost/I
35	502.3_24.4	0.14	0.21	0.03	Orange compost/I
36	502.4_24.4	0.13	0.24	0.08	Orange compost/I
37	502.5_24.4	1.63	0.06	0.08	Orange compost/I
38	502.1_2.5	1.59	0.20	0.09	Orange compost/I
39	502.2_2.5	1.33	0.22	0.08	Orange compost/I
40	502.3_2.5	0.33	0.42	0.03	Orange compost/I
41	502.4_2.5	0.47	0.01	0.08	Orange compost/I
42	502.5_2.5	0.43	0.02	0.08	Orange compost/I
43	502.1_8.5	0.80	0.13	0.09	Orange compost/I
44	502.2_8.5	0.47	<0.01	0.08	Orange compost/I
45	502.3_8.5	0.63	0.27	0.03	Orange compost/I
46	502.4_8.5	0.77	<0.01	0.03	Orange compost/I
47	502.5_8.5	1.59	0.05	0.08	Orange compost/I
48	502.1_24.5	1.14	0.05	0.09	Orange compost/I
49	502.2_24.5	0.64	0.03	0.08	Orange compost/I
50	502.3_24.5	3.91*	1.27*	0.09	Orange compost/I
51	502.4_24.5	0.63	0.16	0.09	Orange compost/I
52	502.5_24.5	0.67	0.38	0.08	Orange compost/I
	Mean EC_50_	0.84 ± 0.11	0.19 ± 0.02	0.06 ± 0.01	
53	WT	0.41	<0.01	0.07	Ref/UK
54	TR_34_ +L98H	2.58	6.60	2.31	Ref/NL
55	TR_34_ +L98H	5.12	4.42	2.31	Ref/UK
56	TR_46_ +Y121F+T289A	4.42	4.37	0.96	Ref/UK

Ref = reference isolates, I = Italy, UK = United Kingdom, NL = The Netherlands.

*not included in calculations of mean EC_50_

### *In vitro* sensitivity to DMIs of *A*. *fumigatus* isolates

Fifty *A*. *fumigatus* isolates from orange-based compost and 2 isolates from orange fruits were investigated for sensitivity to the three DMI fungicides, voriconazole, posaconazole and imazalil (Table 2). The intrinsic antifungal activity was highest for imazalil (mean EC_50_ = 0.06±0.01 mg/l, range <0.01–0.09 mg/l), followed by posaconazole (mean EC_50_ = 0.19±0.02 mg/l, range <0.01–0.72 mg/l), and voriconazole (mean EC_50_ = 0.84±0.11 mg/l, range 0.11–0.70 mg/l). The sensitivity range between the most and least sensitive isolate was 6-fold for voriconazole, 9-fold for imazalil, and 72-fold for posaconazole (Table 2).

Fifty-two *A*. *fumigatus* isolates were fully sensitive to the tested DMI fungicides, with exception of one isolate (502.3_24.5) in combination with two mecidal triazoles (voriconazole and posaconazole). This isolate was the only one among five isolates from the same (and the last) sampling date with an increased EC_50_ value. Two isolates (502.2_24.4, and 502.2_24.5) that showed polymorphisms at aa positions 46, 120, 172 and 427 were also fully sensitive to the three tested fungicides.

**Fig 3:**
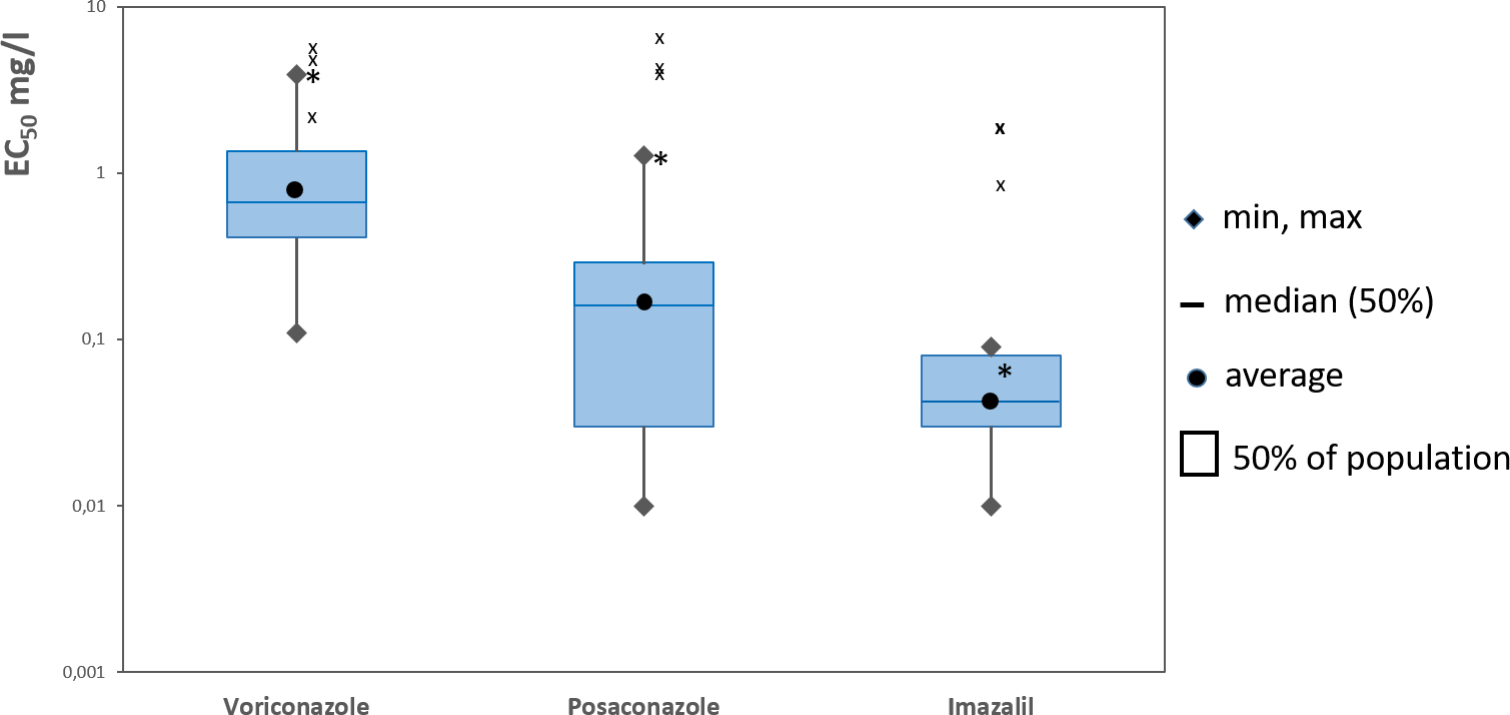
Sensitivity distribution (EC_50_) of *Aspergillus fumigatus* isolates from orange-based compost to three DMIs. Three reference resistant isolates (TR_34_+L98H from UK, TR_46_+Y121F+T289A from UK, and TR_34_+L98H from NL) are included and shown by cross. Orange-based compost isolate 502.3_24.5 with reduced sensitivity to voriconazole and posaconazole is shown by star.

The ‘box-and-whiskers’ plots including one wild-type and three resistant reference isolates showed important sensitivity variations in the 50% box of population for posaconazole, and high maximum whiskers values for voriconazole and posaconazole. The sensitivity of the orange-based compost isolate 502.3_24.5 was clearly outside the 50% box for voriconazole and posaconazole. The highest whiskers value was observed for posaconazole. The most uniform sensitivity distribution taking in consideration all calculated parameters was found for imazalil (Fig 3).

### Molecular characterization of the *cyp51A* coding gene sequence

**Fig 4:**
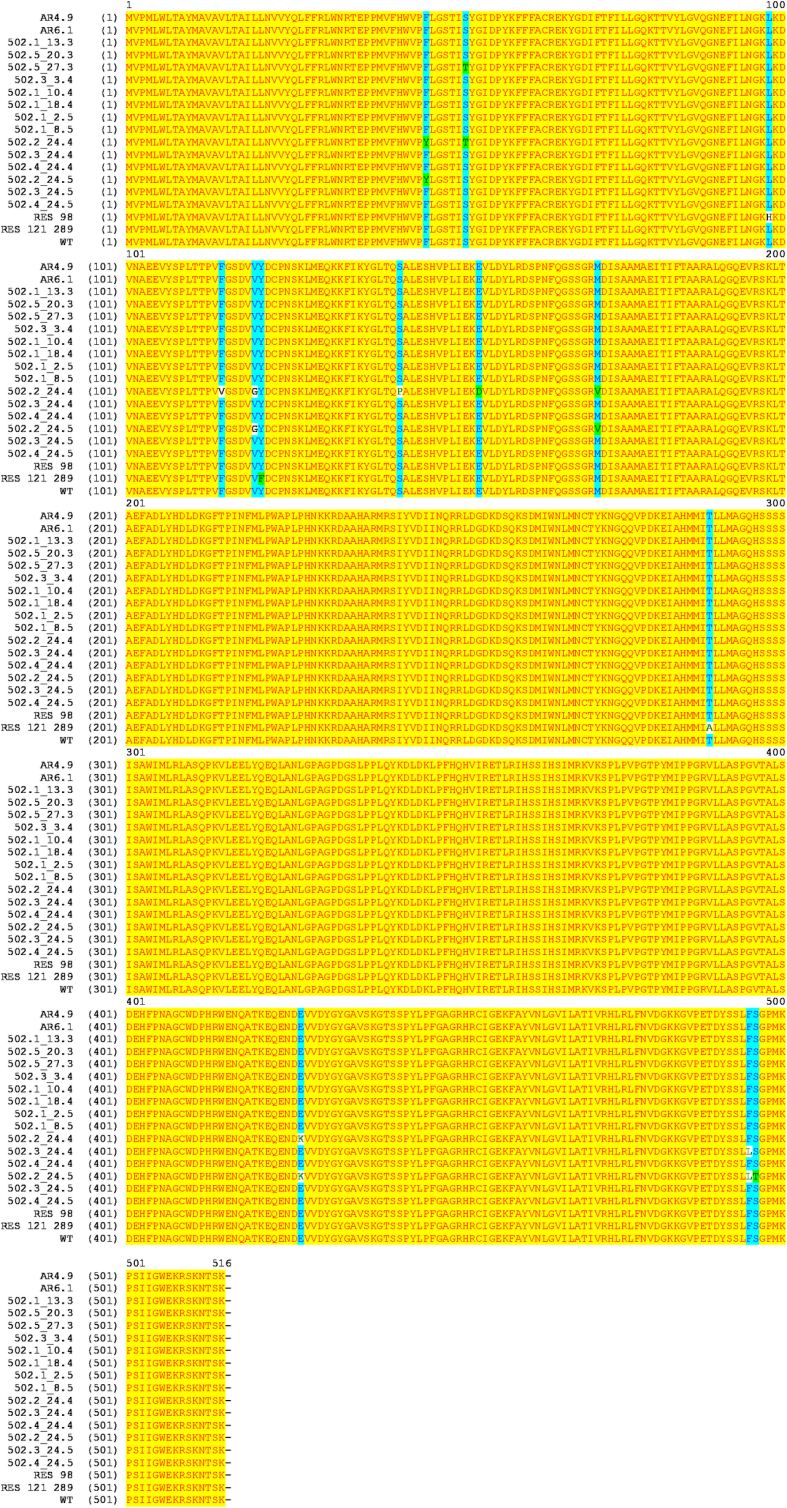
Cyp51A amino acid sequence multiple alignment of 14 isolates of *A*. *fumigatus* from orange-based compost and 2 isolates from oranges. Two reference DMI resistant isolates (L98H, and Y121F+T289A) and wild-type reference (WT) are included in analysis. Background amino acid colour: yellow = identical amino acids; blue = conservative amino acids; green = block of similar amino acids; white = non similar amino acids.

Out of 14 A. *fumigatus* isolates from orange-based compost, two isolates (502.2_24.4 and 502.2_24.5) belonging to two different composting periods (24 April and 24 May) showed 4 shared *cyp51A* polymorphisms: F46Y, V120G, M172V, and E427K (Fig 4). The isolates 502.5_27.3 and 502.2_24.4 shared the mutation S52T, and the isolates 502.3_24.4 and 502.2_24.5 the mutation F495L. Single aa substitutions were also found in isolate 502.2_24.4 at positions 115 (F to V), 142 (S to P) and 154 (E to D), and in the isolate 502.2_24.5 at position 496 (S to T). On the other hand, 10 other isolates did not show any polymorphisms, including the isolate 502.3_24.5 that showed reduced sensitivity to voriconazole and posaconazole (Fig 4). Also, the two *A*. *fumigatus* isolates from orange fruits (AR4.9 and AR6.1) had no aa substitutions. Mutations known to be responsible in DMI resistance (L98H, Y121F, and T289A) were present in the resistant reference isolates but not in any of the sequenced *A*. *fumigatus* isolates from compost, and orange fruits.

Moreover, all isolates from this and previous studies [4,5] were aligned in the coding region of the *cyp51A* gene (515 aa). Phylogenetic analyses grouped the isolates in two clusters (S2 Fig). The main cluster contained all orange-based compost isolates, WT and resistant reference isolates, while the second cluster included the compost isolates with a higher number of *cyp51A* polymorphisms.

## Discussion

*A*. *fumigatus* is not a plant pathogen and does not invade living plant cells. However, it can grow easily as saprophyte in decaying organic matter. Presence of *A*. *fumigatus* has also been associated with the spoilage and loss of citrus fruits during storage [33]. Several *Aspergillus* spp. have been reported as epiphytes on the peel of citrus fruits [34,35]. During the composting process of orange fruits *A*. *fumigatus* population was found to increase a 100-fold already after 1 week, confirming the degradation power for organic matter at high temperatures of this thermo-tolerant fungus [7]. The maturing phase of composting in which the temperatures were still high and pH changed from acidic to slightly basic, caused a further increase of fungal growth, demonstrating that degradation of orange fruits favoured the presence and abundance of *A*. *fumigatus* in compost. Similarly, abundance of *A*. *fumigatus* increased tremendously in corn silage already one week after silo opening [36]. Therefore, it can be expected that in all organic substrates undergoing a thermophilic phase during degradation (eliminating a big portion of the microbial populations) *A*. *fumigatus* may be present in rather high quantities. This represents a quite high health risk especially for immunocompromised people and those who are sensitive to allergic reactions. In addition, the fungus may contaminate the organic substrates through the production of mycotoxic compounds such as fumigacin [14].

*N*. *fumigata*, the teleomorph (sexual) stage of *A*. *fumigatus*, is probably rare under natural conditions and only recently discovered [37]. Genes associated with the expression of both mating types are present in the genome of *A*. *fumigatus*. Mating type 1 is considered to be associated to higher invasiveness of *A*. *fumigatus* in patients, whereas mating type 2 is more frequent in environmental populations [38]. In our study, none of the isolates expressed simultaneously both mating types, most of them belonged to mating type 2, including those isolated from orange peels, whereas mating type 1 evolved towards the end of the composting process. This pattern demonstrates that also mating type 1 isolates can develop in environmental populations even if there was a predominance of mating type 2 at the beginning of the process.

The analysis of the SSR genetic diversity showed that the five isolates from the final composting process belonged to mating type 1 also clustered together with three DMI resistant reference isolates in cluster II. Moreover, many of the cluster I isolates, including one other mating type 1 isolate harbored several polymorphisms in *cyp51A* gene but were sensitive to the three tested fungicides, suggesting that, in the presence of low azoles concentrations, composting of orange fruits would not select for azole-resistant *A*. *fumigatus* genotypes nor for the well known mutations coding for triazole resistance (L98H, Y121F, T289A).

*A*. *fumigatus* isolates from orange-based compost (this study) exhibited moderate SSR polymorphisms supporting microsatellite genotypic results of other environmental populations [39] and commercial compost [5]. Furthermore, it was not possible to separate DMI-sensitive and DMI-resistant strains. Although three resistant reference strains grouped together in the same sub-cluster, they were together with a wild-type reference isolate. Thus, SSR genotyping is not related to fungicide sensitivity as was already observed in previous studies [5,30,39]. However, clustering might be related to the geographic origin as shown earlier by Santoro *et al*. [5] and to a specific composting phase, since all isolates from mature compost were grouped together in one cluster, while isolates from the earlier composting phases were in another cluster. Our data also showed that SSR markers were polymorphic even within the same sample suggesting a high genetic drift or frequent mutations happening from one generation to the next. Also, *A*. *fumigatus* population may not simply grow clonally, but may undergo continuous sexual or parasexual recombination as was already reported earlier [40].

Few *A*. *fumigatus* isolates in this study showed *cyp51A* polymorphisms but were sensitive to DMIs as was reported already in environmental isolates: F46Y from soil and indoor/outdoor hospital environment [3,28]; S52T from seeds and compost [3,5]; F115V, V120G, S142P and E145Q from compost [5]; M172V from soil and indoor/outdoor hospital environment [3,28]; E427K from soil, indoor/outdoor hospital environment, and compost [3,4,28]; and F495L from indoor/outdoor hospital environment [28]. These polymorphisms were reported also in sensitive clinical isolates (reviewed by Stensvold *et al*. [41]). None of the orange-based compost isolate in this study owned any of the *cyp51A* mutations related to DMI resistance, similarly to our previous findings on compost and biochar isolates with different geographical origins (Italy, Spain, Hungary, The Netherlands, Germany and United Kingdom) [4,5]. The cyp51A amino acid changes found in this study more likely present particular genotypes related to specific developmental stage (soil, compost) rather than specific locations. Taking all isolates from this and earlier studies [4,5] together, there was no *cyp51A* clustering pattern according to geographic origin (countries) (see S2 Fig). On the other hand, the isolate 502.3_24.5 expressed reduced sensitivity to both medical triazoles, voriconazole and posaconazole (although no cyp51A mutation was detected), but was fully sensitive to imazalil. This may suggest that this isolate either has been selected preferentially by the medical triazoles (preferential selection is known within DMIs in certain plant pathogens, [42]), or could express resistance mechanisms that are specific for the two medical triazoles (e.g. ABC pumps).

It is still under debate whether DMI resistance in *A*. *fumigatus* has primarily medical origin (due to medical/veterinary DMI treatments of aspergilloses) or environmental and/or agricultural origin (due to DMI applications against plant pathogens and in material protection). Our findings so far suggested that *A*. *fumigatus* of environmental origin is not a source for *cyp51A* mutations associated with DMI resistance. The same findings were observed for environmental *A*. *fumigatus* isolates from compost and biochar in our previous studies [4,5] and in other similar investigations [43–45]. DMI resistance observed in *A*. *fumigatus* isolates of environmental and clinical origin in other studies [6,30–32,39,46–48] might be associated to much higher azole concentrations in the substrate (and subsequently higher selection pressure) or to certain geographic locations.

Composting obviously presents a very favourable niche for *A*. *fumigatus* to grow and multiply, however the process itself might be disadvantageous for resistance evolution probably due to degradation or adsorption of fungicides in the organic substrate [49]. Since imazalil is not as persistent in soil environment (dissipation half-life (DT_50_): 30–170 days, [50]) and compost at low pH (DT_50_: 55–120 days, [51, 52]) as certain agricultural triazoles, it can be assumed that its residue quantities in the orange-based compost are not sufficient for a possible selection of resistant individuals. Consequently, composting orange fruits would allow *A*. *fumigatus* to reproduce, but not to select azole-resistant genotypes. Furthermore, our study demonstrates a high genetic diversity in *A*. *fumigatus* isolates even when deriving from the same environmental sample, suggesting frequent sexual or para-sexual recombination events within the population. However, it was not possible to genetically distinguish sensitive environmental from resistant reference isolates because they were within the same clusters. Finally, more emphasize should be given to the presence of *A*. *fumigatus* in compost in order to achieve better safety precautions for people in close contact with compost (e.g. compost operators).

## Conclusions

In the present study, the abundance, genetic diversity and DMI sensitivity of *A*. *fumigatus* was evaluated during the composting process of orange fruits. Although composting presents a very suitable niche for *A*. *fumigatus* grow, it may be assumed that imazalil residue concentrations are not sufficient to select resistant genotypes during orange-based composting.

## Supporting information

S1 Fig Mating type identification of 25 *Aspergillus fumigatus* isolates by multiplex PCR assay. The amplicons of 834 bp for MAT1-1 or 438 bp for MAT1-2 are indicated. L = GelPilot 1 kb Plus Ladder (Qiagen), 1 = 502.1_18.4, 2 = 502.2_18.4, 3 = 502.3_18.4, 4 = 502.4_18.4, 5 = 502.5_18.4, 6 = 502.1_24.4, 7 = 502.2_24.4, 8 = 502.3_24.4, 9 = 502.4_24.4, 10 = 502.5_24.4, 11 = 502.1_2.5, 12 = 502.2_2.5, 13 = 502.3_2.5, 14 = 502.4_2.5, 15 = 502.5_2.5, 16 = 502.1_8.5, 17 = 502.2_8.5, 18 = 502.3_8.5, 19 = 502.4_8.5, 20 = 502.5_8.5, 21 = 502.1_24.5, 22 = 502.2_24.5, 23 = 502.3_24.5, 24 = 502.4_24.5, 25 = 502.5_24.5, C = negative control.
(TIF)

S2 Fig Phylogenetic relationship of *Aspergillus fumigatus* isolates from orange-based, green and brown composts, and biochars of different origins: Spanish (S), Hungarian (H), Dutch (NL), British (UK) and Italian (I) isolates based on the cyp51A amino acid sequence inferred by Neighbour-joining analysis. Bootstrap analysis is supported with 1000 replications. The isolate 502.3_24.5 with reduced sensitivity to voriconazole and posaconazole is shown in italics. Cyp51A amino acid sequences are from this study, Francheschini et al. [4], Santoro et al. [5]. Reference resistant isolates are also included and shown in bold: ITZ.86_Rc, Snelders et al. [28]; 11_0087A_Re, Prigitano et al. [31]; 14_Re, Van der Linden et al. [30]; 98 Rc, Van Ingen et al. [32]. Wild-type AF338659 –WT, Mellado et al. [13].
(TIF)
