# Correction: Molecular characterization and sensitivity to demethylation inhibitor fungicides of *Aspergillus fumigatus* from orange-based compost

**DOI:** 10.1371/journal.pone.0202835

**Published:** 2018-08-16

**Authors:** Massimo Pugliese, Slavica Matić, Sanila Prethi, Ulrich Gisi, Maria Lodovica Gullino

There is an error in Table 1. The values in column 3 "Accession no. (ITS)" are incorrect. Please see the corrected Table 1 here.

**Table 1:** List of *A*. *fumigatus* isolates selected for sensitivity assays with accession numbers and mating type.

Code	Sampling date	Accession no. (ITS)	Accession no. (*cyp51A*)	Mating type	
				1	2
AR4.9	28 February 2017	MG976901	MH026061		+
AR6.1		MG976902	MH026062		+
502.1_13.3	13 March 2017	MG970367	MH026063		+
502.2_13.3		MG970368			+
502.3_13.3		MG970369			+
502.4_13.3		MG970370			+
502.5_13.3		MG970371			+
502.1_20.3	20 March 2017	MG970372			+
502.2_20.3		MG970373			+
502.3_20.3		MG970374			+
502.4_20.3		MG970375			+
502.5_20.3		MG970376	MH026064		+
502.1_27.3	27 March 2017	MG970377			+
502.2_27.3		MG970378			+
502.3_27.3		MG970379			+
502.4_27.3		MG970380			+
502.5_27.3		MG970381	MH026065		+
502.1_3.4	3 April 2017	MG970382			+
502.2_3.4		MG970383			+
502.3_3.4		MG970384	MH026066		+
502.4_3.4		MG970385			+
502.5_3.4		MG970386			+
502.1_10.4	10 April 2017	MG970387	MH026067		+
502.2_10.4		MG970388			+
502.3_10.4		MG970389			+
502.4_10.4		MG970390			+
502.5_10.4		MG970391			+
502.1_18.4	18 April 2017	MG970392	MH026068		+
502.2_18.4		MG970393			+
502.3_18.4		MG970394			+
502.4_18.4		MG970395			+
502.5_18.4		MG970396			+
502.1_24.4	24 April 2017	MG970397			+
502.2_24.4		MG970398	MH026069	+	
502.3_24.4		MG970399	MH026070		+
502.4_24.4		MG970400	MH026071		+
502.5_24.4		MG970401			+
502.1_2.5	2 May 2017	MG970402	MH026072		+
502.2_2.5		MG970403			+
502.3_2.5		MG970404			+
502.4_2.5		MG970405			+
502.5_2.5		MG970406			+
502.1_8.5	8 May 2017	MG970407	MH026073		+
502.2_8.5		MG970408			+
502.3_8.5		MG970409			+
502.4_8.5		MG970410			+
502.5_8.5		MG970411			+
502.1_24.5	24 May 2017	MG970412		+	
502.2_24.5		MG970413	MH026074	+	
502.3_24.5		MG970414	MH026075	+	
502.4_24.5		MG970415	MH026076	+	
502.5_24.5		MG970416		+	
