# Supplementary figures and images for: Molecular characterization and sensitivity to demethylation inhibitor fungicides of Aspergillus fumigatus from orange-based compost
Source: PLoS One. 2018 Jul 12;13(7):e0200569. doi: 10.1371/journal.pone.0200569 (PMC6042770; doi:10.1371/journal.pone.0200569)

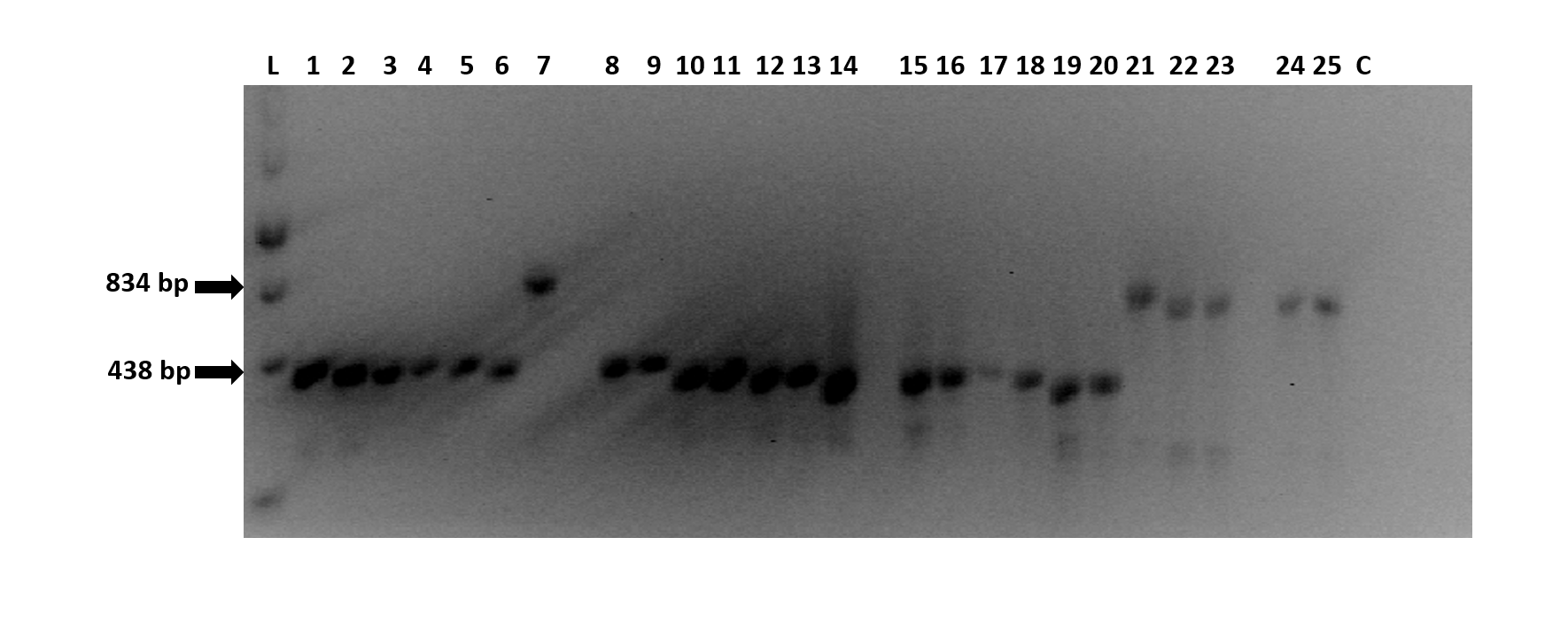

Supplement: S1 Fig — The amplicons of 834 bp for MAT1-1 or 438 bp for MAT1-2 are indicated. L = GelPilot 1 kb Plus Ladder (Qiagen), 1 = 502.1_18.4, 2 = 502.2_18.4, 3 = 502.3_18.4, 4 = 502.4_18.4, 5 = 502.5_18.4, 6 = 502.1_24.4, 7 = 502.2_24.4, 8 = 502.3_24.4, 9 = 502.4_24.4, 10 = 502.5_24.4, 11 = 502.1_2.5, 12 = 502.2_2.5, 13 = 502.3_2.5, 14 = 502.4_2.5, 15 = 502.5_2.5, 16 = 502.1_8.5, 17 = 502.2_8.5, 18 = 502.3_8.5, 19 = 502.4_8.5, 20 = 502.5_8.5, 21 = 502.1_24.5, 22 = 502.2_24.5, 23 = 502.3_24.5, 24 = 502.4_24.5, 25 = 502.5_24.5, C = negative control. (TIF) [file pone.0200569.s001.tif]

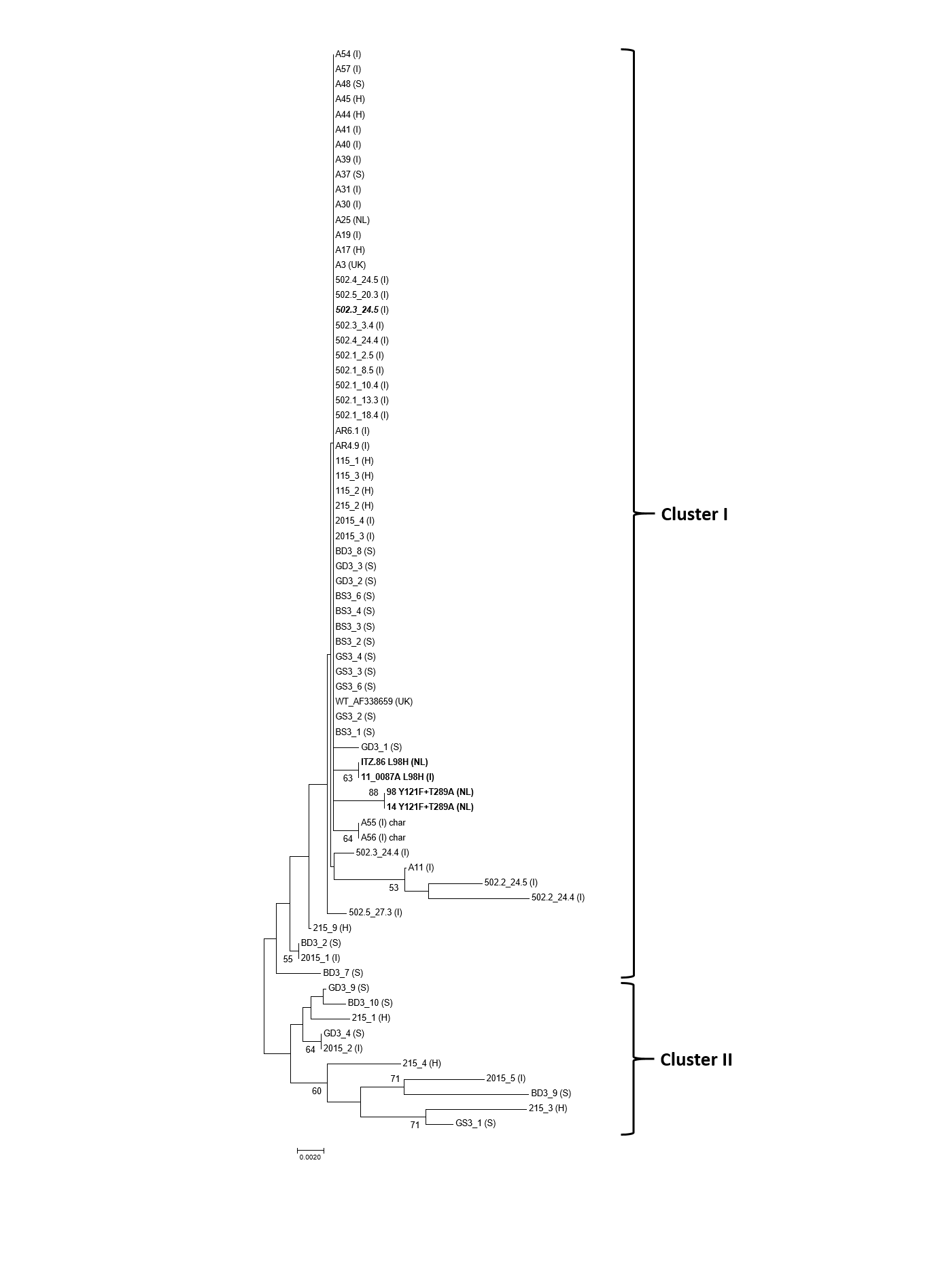

Supplement: S2 Fig — Bootstrap analysis is supported with 1000 replications. The isolate 502.3_24.5 with reduced sensitivity to voriconazole and posaconazole is shown in italics. Cyp51A amino acid sequences are from this study, Francheschini et al. [4], Santoro et al. [5]. Reference resistant isolates are also included and shown in bold: ITZ.86_Rc, Snelders et al. [28]; 11_0087A_Re, Prigitano et al. [31]; 14_Re, Van der Linden et al. [30]; 98 Rc, Van Ingen et al. [32]. Wild-type AF338659 –WT, Mellado et al. [13]. (TIF) [file pone.0200569.s002.tif]
